# Investigating the Salivary Biomarker Profile in Obesity: A Systematic Review

**DOI:** 10.1007/s13679-025-00618-y

**Published:** 2025-03-28

**Authors:** H. AL Habobe, R. H.H. Pieters, F. J. Bikker

**Affiliations:** 1 Research Group Innovative Testing in Life Sciences and Chemistry, Research Centre for Healthy and Sustainable Living, University of Applied Sciences Utrecht, Utrecht, The Netherlands; 2 Institute for Risk Assessment Sciences, Faculty of Veterinary Medicine, Utrecht University, Utrecht, The Netherlands; 3 Department of Oral Biochemistry, Academic Centre for Dentistry Amsterdam (ACTA), University of Amsterdam and VU University Amsterdam, Amsterdam, The Netherlands

**Keywords:** Obesity, Saliva, Biomarkers, Metabolic syndrome, MUO

## Abstract

**Purpose of Review:**

This systematic review aims to map the existing literature on salivary biomarkers in adults with metabolically unhealthy obesity (MUO), identify key biomarkers associated with this high-risk group, and highlight areas requiring further research to advance this emerging field.

**Recent Findings:**

Obesity is characterized by an abnormal accumulation of body fat and chronic inflammation. However, not all individuals with obesity experience metabolic dysfunction. This review focuses on MUO, which is strongly linked to metabolic disorders such as insulin resistance, cardiovascular disease, type 2 diabetes, and systemic inflammation. Linking MUO and salivary biomarkers may enhance our understanding of how systemic health influences salivary composition and could enable the early identification of high-risk individuals through non-invasive saliva testing. This review synthesized findings from recent studies and identified key salivary biomarkers consistently elevated in individuals with MUO, including 8-OHdG, IL-6, IL-8, resistin, TNFR1, PTX-3, AEA, OEA, TNF-α, and sICAM-1. These biomarkers are associated with inflammation, oxidative stress, and metabolic dysregulation. The majority of studies utilized cross-sectional designs and used various saliva collection methods.

**Summary:**

Salivary biomarkers hold promise as non-invasive indicators of obesity-related metabolic dysfunction, particularly in MUO. However, their clinical diagnostic utility remains uncertain due to heterogeneity in study designs, a lack of biomarker validation, and limited longitudinal studies. Further research is needed to establish their bona fide diagnostic potential.

**Supplementary Information:**

The online version contains supplementary material available at 10.1007/s13679-025-00618-y.

## Introduction

Obesity is a complex and multifactorial condition that has emerged as a major global health challenge that affect millions of individuals worldwide. It is characterized by an abnormal accumulation of body fat and chronic inflammation, leading to significant health risks [1–3]. Obesity is associated with numerous chronic diseases, including type 2 diabetes, cardiovascular disease, liver disease, periodontitis and certain cancers [4–8]. The World Health Organization (WHO) identifies obesity as a global problem driven by an imbalance between energy intake and expenditure [9]. However, not all individuals with obesity experience metabolic complications. A distinct subgroup, referred to as individuals with metabolically unhealthy obesity (MUO), exhibits metabolic disturbances such as insulin resistance, chronic inflammation, and dyslipidemia. MUO is strongly associated with an increased risk of type 2 diabetes and all-cause mortality [10–12]. Despite considerable efforts and ongoing interventions, its prevalence continues to rise, highlighting the need for effective treatment methods and monitoring.

Traditionally, the diagnosis and management of obesity-related conditions have relied on anthropometric measurements such as Body Mass Index (BMI), waist circumference (WC), fat mass (FM) and fat-free mass (FFM) [13]. However, these indices do not always provide an accurate picture of metabolic risk. For example, studies have shown that BMI alone cannot differentiate between metabolically healthy obesity (MHO) and MUO, as individuals with the same BMI may have vastly different metabolic profiles [14]. This is partly because BMI does not account for fat distribution, particularly central obesity, which is characterized by the excessive accumulation of visceral fat and plays a critical role in MUO as a key determinant of metabolic dysfunction [15]. Consequently, reliance on BMI-based indices may lead to the misclassification of individuals as obese. At one end of the spectrum, some individuals with obesity seem to be protected from the typical metabolic complications. Conversely, at the other end, individuals with normal weight may experience metabolic disorders associated with obesity [14]. This suggests that obesity assessment should extend beyond anthropometric indices and incorporate biochemical markers that reflect metabolic health. Therefore, understanding the pathophysiological mechanisms underlying body weight changes at the molecular level is crucial for accurate diagnosis, as it provides valuable insight into obesity-related metabolic dysregulations and associated health risks.

Although blood samples have traditionally been used to study pathophysiology, interest has recently grown in using saliva as a non-invasive medium for biomarker analysis and disease monitoring [16–17]. Saliva offers several advantages over blood, including cost-effectiveness, ease of collection, minimized patient discomfort, and the ability to collect multiple samples without the need for specialized personnel or equipment [18]. Like blood, saliva contains a variety of biomarkers including ions, proteins, hormones, fatty acids, and vitamins [19]. As a result, salivary biomarkers have gained considerable attention for their potential to provide insights into the pathophysiological processes underlying obesity and its related complications. Despite this growing interest, research on salivary biomarkers in the context of obesity remains fragmented [20–22], with various studies examining different biomarkers without offering a cohesive or comprehensive synthesis of the findings.

This systematic review aimed to comprehensively explore the existing literature on salivary biomarkers in adults with MUO. Given that MUO is strongly associated with metabolic disorders such as insulin resistance, type 2 diabetes, and chronic inflammation, the identification of salivary biomarkers in this population may provide a non-invasive tool for the early detection of metabolic dysfunction. In this review, we systematically mapped the studied salivary biomarkers and identified potential key candidates that could serve as early indicators of MUO. Furthermore, we identified research gaps and areas requiring further investigation to advance the reliability and clinical applicability of salivary biomarkers for the early detection of MUO.

## Methods

### Protocol Registration

This systematic review was registered in the PROSPERO International Prospective Register of Systematic Reviews (CRD42024602338) and was structured in accordance with the Preferred Reporting Items for Systematic Reviews and Meta-Analyses (PRISMA) guidelines [23].

### Study Design

This systematic review was designed to identify and synthesize the existing body of scientific literature on salivary biomarkers found in adults with MUO.

*Research question*: *Which salivary biomarkers have been investigated and associated with metabolically unhealthy obesity in adults in the existing literature?*

The review focused on observational studies, including cohort, cross-sectional, and case-control studies, as well as clinical trials that examined the association between salivary biomarkers and obesity. To be included, studies were required to meet specific inclusion criteria based on the PICO guidelines [24].

*Population (P): Adults aged 18 years and older with obesity (BMI ≥ 30 kg/m²).*

*Intervention (I): Analysis of salivary biomarkers.*

*Comparison (C): Adults aged 18 years and older without obesity (BMI 18.5–24.99 kg/m²)*,* serving as a control group.*

*Outcome (O): Description of identified and characterized salivary biomarkers associated with obesity.*

### Search Strategy

The literature search was conducted between July 2024 and December 2024, covering studies published from January 2000 to December 2024. A comprehensive search strategy was conducted to identify publications that studied salivary biomarkers in adults with obesity. Five major electronic databases were searched: PubMed, Embase, Web of Science, Scopus and ScienceDirect. The search strategy was designed to capture a broad range of studies by including the key terms: “Saliva” AND “Biomarkers” AND “Obesity” combined with related terms using the OR operator.

Key terms and related terms:

*Saliva*: Saliva, salivary.

*Biomarkers*: Biomarker(s), marker biological, biological marker(s), biologic marker(s), markers biologic, biological factor(s), immune marker(s), marker immune, immunologic marker(s), markers immunologic, clinical marker(s), markers laboratory, laboratory marker, clinical markers, biochemical marker(s), viral marker(s), inflammatory marker(s), viral markers, marker viral, biochemical marker, marker biochemical.

*Obesity*: Obesity, abdominal obesity, abdominal obesities, obesities abdominal, visceral obesity, visceral obesities, obesity visceral, obesities visceral, central obesity, general obesity, central obesities, obesities central, obesity central, morbid obesity, morbid obesities, severe obesity, severe obesities, pediatric obesity, body mass index (BMI), overweight, obesity abdominal, type 2 diabetes, diabetes mellitus type 2, T2DM, insulin resistance, insulin-resistant, hyperinsulinemia, insulin sensitivity, fatty liver, steatohepatitis, visceral steatosis, liver steatosis, dyslipidemia(s), dyslipoproteinemia(s), hyperlipidemia(s), hyperlipemia(s), hypercholesterolemia, cholesterol, high cholesterol level(s).

Additionally, truncations, quotation marks, field tags, MeSH (Medical Subject Headings) terms, and the tiab (title/abstract) field were applied as appropriate to refine and optimize the search. The search was restricted to studies published between 2000 and 2024, focusing on human studies written in English. To ensure the relevance of the studies, filters (NOT-operator) were applied to exclude research involving adolescents, children, and specific populations such as pregnancy, as well as studies not directly related to salivary biomarkers or obesity.

Boolean operators were applied as follows:

- **AND** to combine major themes (e.g., “saliva” **AND** “abdominal obesity”).
- **OR** to include alternative terms (e.g., “obesity” **OR** “central obesity”).
- **NOT** to exclude irrelevant studies (e.g., “NOT children” to focus on adult populations).

Additionally, studies that were not peer-reviewed, such as conference abstracts, literature reviews, case reports and dissertations, were excluded. Inclusion and exclusion criteria are presented in Table 1.

**Table 1:** Inclusion and exclusion criteria used for screening the title/abstract

Inclusion criteria	Exclusion criteria
• **Population**: Studies involving adults (18 years and older) with obesity (or BMI ≥ 30 kg/m^2^). • **Intervention**: Studies analyzing salivary biomarkers. • **Comparison**: studies including a control group without obesity. • **Outcome**: Studies reporting on types of salivary biomarkers associated with obesity. • **Study Status**: Published, full text articles. • **Language**: Studies written in English. • **Study Design**: Observational studies (cohort, cross-sectional, case-control) and clinical trials.	• **Population**: Studies focusing on children or adolescents (under 18 years); studies on, or including specific populations such as pregnant women. • **Intervention**: Studies not measuring salivary biomarkers; including those using blood and urine only. • **Outcome**: Studies not providing information on the association between salivary biomarkers and obesity/BMI. Also, studies that measure behavioral, psychological, or purely demographic factors without biological analysis in saliva. • **Study Status**: Unpublished articles such as conference abstracts, dissertations, or non-peer-reviewed preprints. • **Language**: Studies written in languages other than English. • **Study Design**: Case reports, letters to the editor, literature reviews, commentaries and opinion articles.

### Selection and Quality Assessment Process

After removing duplicates, the articles were screened by one author (H.AL.) for relevance based on their titles and abstracts. Following this initial screening, the selected articles underwent full-text review, conducted by three authors (H.AL., R.P., and F.B.). These studies were then further assessed for eligibility and potential risk of bias using the Quality Assessment Tool for Observational Cohort and Cross-Sectional Studies developed by NIH (National Heart, Lung, and Blood Institute) [25], (Table 2). Any disagreements or uncertainties were resolved through discussion. A single author (H.AL.) extracted the data, which was further verified by the other two authors R.P. and F.B..

**Table 2:** Eligibility assessment of the full texts using the quality assessment tool for observational cohort and Cross-Sectional studies developed by NIH

Questions	Al-Ameri et al. (2024)	Al-Rawi et al. (2017)	Benedix et al. (2011)	Cheprasova et al. (2023)	Fejfer et al. (2017)	Junqueira et al. (2023)	Lamy et al. (2015)	Lehmann et al. (2018)
Was the research question or objective in this paper clearly stated?	Yes	Yes	Yes	Yes	Yes	Yes	Yes	Yes
Was the study population clearly specified and defined?	Yes	Yes	Yes	Yes	Yes	Yes	Yes	Yes
Was the participation rate of eligible persons at least 50%?	No	Yes	No	Yes	Yes	Yes	Yes	No
Were all the subjects selected or recruited from the same or similar populations (including the same time period)? Were inclusion and exclusion criteria for being in the study prespecified and applied uniformly to all participants?	Yes	Yes	Yes	Yes	Yes	Yes	Yes	Yes
Was a sample size justification, power description, or variance and effect estimates provided?	Yes	No	No	No	No	No	No	No
For the analyses in this paper, were the exposure(s) of interest measured prior to the outcome(s) being measured?	No	No	No	No	No	No	No	No
Was the timeframe sufficient so that one could reasonably expect to see an association between exposure and outcome if it existed?	No	No	No	No	No	No	No	No
For exposures that can vary in amount or level, did the study examine different levels of the exposure as related to the outcome (e.g., categories of exposure, or exposure measured as continuous variable)?	NA	NA	NA	NA	NA	NA	NA	NA
Were the exposure measures (independent variables) clearly defined, valid, reliable, and implemented consistently across all study participants?	NA	NA	NA	NA	NA	NA	NA	NA
Was the exposure(s) assessed more than once over time?	NA	NA	NA	NA	NA	NA	NA	NA
Were the outcome measures (dependent variables) clearly defined, valid, reliable, and implemented consistently across all study participants?	Yes	Yes	Yes	Yes	Yes	Yes	Yes	Yes
Were the outcome assessors blinded to the exposure status of participants?	Yes	Yes	Yes	Yes	Yes	Yes	Yes	Yes
Was loss to follow-up after baseline 20% or less?	NA	NA	NA	NA	NA	NA	NA	NA
Were key potential confounding variables measured and adjusted statistically for their impact on the relationship between exposure(s) and outcome(s)?	Yes	Yes	Yes	Yes	Yes	Yes	Yes	Yes
**Total score**	**Fair**	**Fair**	**Fair**	**Fair**	**Fair**	**Fair**	**Fair**	**Fair**
**Questions**	**Lehmann et al. (2020)**	**Matias et al. (2012)**	**Nigro et al. (2015)**	**Öngöz et al. (2016)**	**Ostrowska et al. (2020)**	**Safabakhsh et al. (2022)**	**Soukup et al. (2012)**	**Zyśk et al. (2023)**
Was the research question or objective in this paper clearly stated?	Yes	Yes	Yes	Yes	Yes	Yes	Yes	Yes
Was the study population clearly specified and defined?	Yes	Yes	Yes	Yes	Yes	Yes	Yes	Yes
Was the participation rate of eligible persons at least 50%?	No	Yes	Yes	Yes	Yes	Yes	Yes	Yes
Were all the subjects selected or recruited from the same or similar populations (including the same time period)? Were inclusion and exclusion criteria for being in the study prespecified and applied uniformly to all participants?	Yes	Yes	Yes	Yes	Yes	Yes	Yes	Yes
Was a sample size justification, power description, or variance and effect estimates provided?	No	No	No	Yes	No	No	No	No
For the analyses in this paper, were the exposure(s) of interest measured prior to the outcome(s) being measured?	No	No	No	No	No	No	No	No
Was the timeframe sufficient so that one could reasonably expect to see an association between exposure and outcome if it existed?	No	No	No	No	No	No	No	No
For exposures that can vary in amount or level, did the study examine different levels of the exposure as related to the outcome (e.g., categories of exposure, or exposure measured as continuous variable)?	NA	NA	NA	NA	NA	NA	NA	NA
Were the exposure measures (independent variables) clearly defined, valid, reliable, and implemented consistently across all study participants?	NA	NA	NA	NA	NA	NA	NA	NA
Was the exposure(s) assessed more than once over time?	NA	NA	NA	NA	NA	NA	NA	NA
Were the outcome measures (dependent variables) clearly defined, valid, reliable, and implemented consistently across all study participants?	Yes	Yes	Yes	Yes	Yes	Yes	Yes	Yes
Were the outcome assessors blinded to the exposure status of participants?	Yes	Yes	Yes	Yes	Yes	Yes	Yes	Yes
Was loss to follow-up after baseline 20% or less?	NA	NA	NA	NA	NA	NA	NA	NA
Were key potential confounding variables measured and adjusted statistically for their impact on the relationship between exposure(s) and outcome(s)?	Yes	Yes	Yes	Yes	Yes	Yes	Yes	Yes
**Total score**	**Fair**	**Fair**	**Fair**	**Fair**	**Fair**	**Fair**	**Fair**	**Fair**

Quality was assessed by the number of “Yes” answers out of 14 questions. The quality was then categorized as **good** (9–11 “Yes” answers), **fair** (5–8 “Yes” answers), or **poor** (≤ 4 “Yes” answers). The following notations were used: **NA** for not applicable, **NR** for not reported, **ND** for not detected, and **CD** for cannot be determined

### Data Extraction

For all included studies, the following data were extracted: author(s), the country where the study was conducted, year of publication, population characteristics (including age, sex, and BMI), the salivary biomarkers analyzed, biomarkers showing significant differences between groups with obesity and control groups, biomarkers significantly correlated with BMI, the saliva collection method, and the analytical methods used.

### Data Synthesis

A qualitative synthesis of the included studies was conducted, as no meta-analysis was performed. Following the approach outlined by Popay and co-workers (2006) [26], the synthesis involved organizing and summarizing the findings from each study, focusing on the types of salivary biomarkers analyzed, their associations with obesity, and the methods used for biomarker analysis. The data were systematically extracted and presented in narrative form, highlighting patterns, trends, and inconsistencies across studies. Special attention was given to the significant differences in biomarker levels between individuals with obesity and control populations, as well as the correlations between specific biomarkers and BMI. This approach allowed for a comprehensive understanding of the current evidence without the need for statistical pooling.

## Results

### Study Selection

**Fig. 1:**
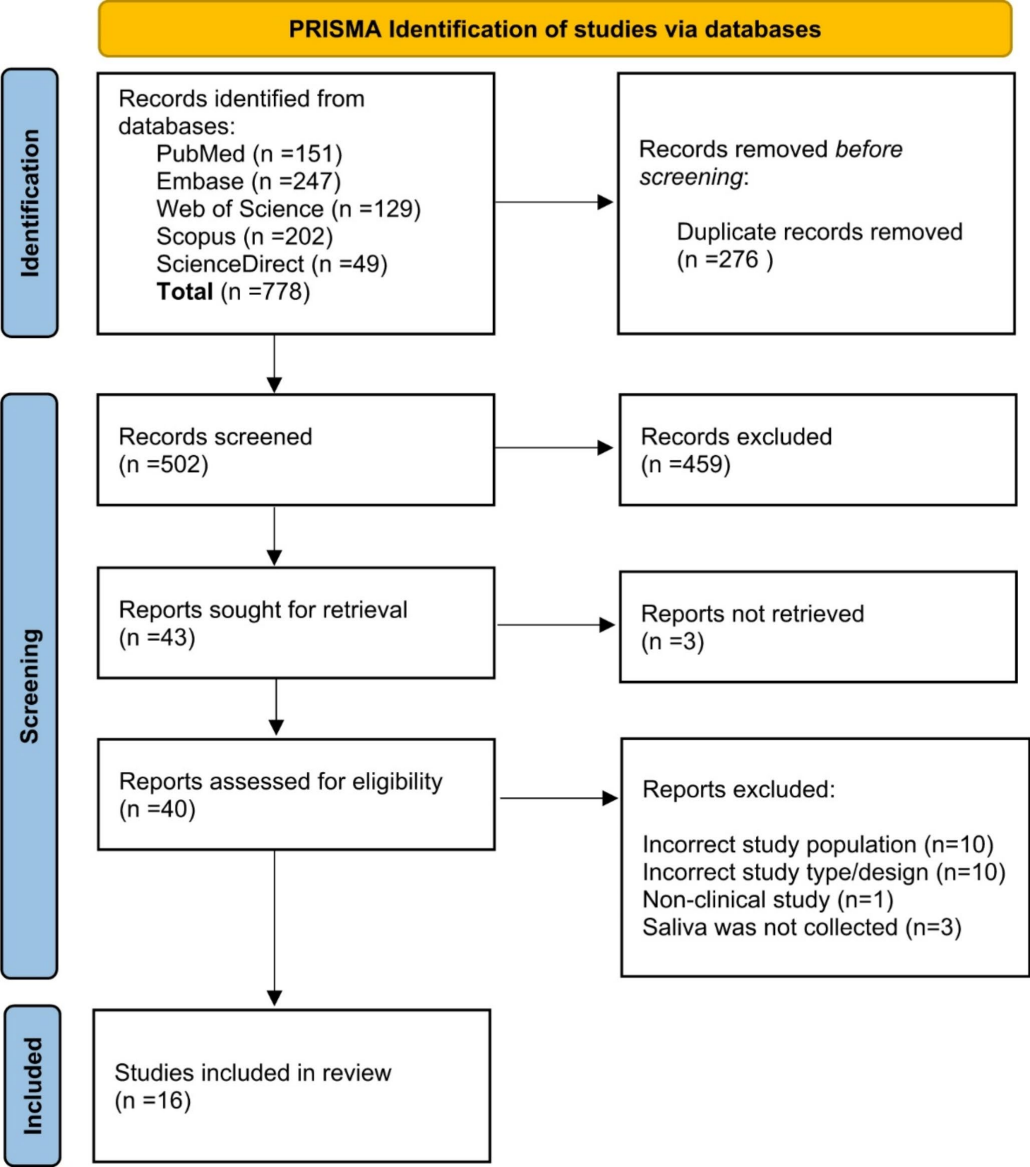
Flowchart diagram of the selection procedure of included articles *(PRISMA guidelines for systematic reviews)*

A total of 778 studies were initially retrieved through a database search, as outlined in the PRISMA diagram in Fig. 1. After removing duplicates, 502 articles remained for title and abstract screening. Of these, 43 articles were selected for full-text review. Three of those were excluded due to the unavailability of the full text. From the remaining 40 articles, 16 articles were included in the synthesis [27–42].

### Quality Assessment of Included Studies

The quality assessment of the included studies is summarized in Table 2. All included studies clearly stated their research questions and objectives, with well-defined study populations. The participation rate of individuals with obesity was at least 50% in most studies, with the exception of four. Of these, three had participation rates above 43% [29, 34, 35], while one study had a participation rate of 33% [27]. Consistent inclusion and exclusion criteria were applied across similar populations in all studies. The majority of studies lacked a justification for sample size, power analysis, or estimates of variance and effect, with the exception of one study [27]. The studies included in the review did not involve interventions or comparable methodologies. Furthermore, none of the studies measured the exposures of interest before assessing the outcomes. Additionally, exposure measures were not reassessed over time, nor were exposure levels analyzed as continuous variables. All outcome measures were clearly defined, valid, and consistently implemented across all studies.

### Population Characteristics of Included Studies

Table 3 provides a summary of the population characteristics across the included studies. The studies span various countries, including Brazil, Portugal, the USA, Russia, Iran, Poland, Turkey, Germany, Italy, France, and the UAE, and utilized different study designs, such as cross-sectional, case-control, and prospective cohort designs. Sample sizes for the groups with obesity and control groups varied considerably across studies, although most had fairly balanced sample sizes between the two groups [28, 30–32, 36–38]. However, some studies had substantial uneven group sizes [27, 29, 33, 34, 41, 42], and some included groups with notably small sample sizes (e.g., less than 13 participants per group) [27, 36, 39]. Thirteen studies included a mixed-sex population, and a few focused exclusively on either male [37] or female participants [33, 39]. Participant ages ranged widely, with mean ages typically between 30 and 50 years, though some studies included broader age ranges [30, 37, 41]. BMI data were provided for both the group with obesity and the control group. The BMI of the control group fell within the normal range (18.5–24.9 kg/m²), whereas the BMI of individuals with obesity consistently exceeded 30 kg/m².

**Table 3:** Summary of population characteristics of included published studies

Author (year)	Location			Sample size (*n*)		Age (years)		BMI (kg/m^2^)	
		**Study design**	**Sex** (Female, Male)	**Group with Obesity**	**Control group**	**Group** **with** **Obesity**	**Control group**	**Group** **with** **Obesity**	**Control** **group**
Al-Ameri et al. (2024)	UAE	Cross-Sectional	mixed	10	20	25.3 ± 5.1	27.2 ± 4.5	35.0 ± 4.8	22.9 ± 3.0
Al-Rawi et al. (2017)	UAE	Cross-Sectional	mixed	26	26	51.1 ± 5.7	47.4 ± 5.0	34.3 ± 3.9	27.1 ± 2.1
Benedix et al. (2011)	Germany	Cross-Sectional	mixed	41	45	42.6 (25–65)*	40.2 (20–58)*	53.1 (35.6–77.2)*	22.7 (18.6–24.8)*
Cheprasova et al. (2023)	Russia	Case Control	mixed	40	40	62.6 ± 1.9	41.0 ± 3.5	33.6 ± 1.2	< 24.99
Fejfer et al. (2017)	Poland	Prospective Cohort	mixed	47	47	44.5 ± 10.5	42.8 ± 13.1	47.1 ± 0.8	20.6 ± 2.3
Junqueira et al. (2023)	Brazil	Cross-Sectional	mixed	14	14	34.0 ± 9.0	32.0 ± 7.0	40.0 ± 7.0	23.0 ± 2.0
Lamy et al. (2015)	Portugal	Cross-Sectional	females	14	11	39.7 ± 3.7	36.1 ± 3.1	44.8 ± 1.8	21.1 ± 0.5
Lehmann et al. (2018)	Poland	Cross-Sectional	mixed	19	25	32.7 ± 6.2	31.1 ± 5.2	38.7 ± 5.4	22.1 ± 3.6
Lehmann et al. (2020)	Poland	Case Control	mixed	59	66	34 (27–40)*	30 (24–36)*	37.1 (32.7–41.4)*	21.8 (19.4–23.9)*
Matias et al. (2012)	France	Cross-Sectional	mixed	12	12	45.0 ± 2.5	45.0 ± 2.5	44.3 ± 3.1	18.5–24.99
Nigro et al. (2015)	Italy	Cross-Sectional	males	27	27	20.0–68.0	20.0–68.0	> 30.0	18.5–24.99
Öngöz Dede et al. (2016)	Turkey	Interventional	mixed	15	15	41.3 ± 6.5	29.6 ± 2.3	36.9 ± 5.2	22.3 ± 1.7
Ostrowska et al. (2020)	Poland	Cross-Sectional	females	10	6	40.0–57.0	32.0–59.0	31.9–36.1	22.5–23.1
Safabakhsh et al. (2022)	Iran	Case Control	mixed	46	46	35.1 ± 10.1	35.6 ± 10.3	> 30.0	18.5–24.99
Soukup et al. (2012)	USA	Cross-Sectional	mixed	27	51	53.7 ± 10.2	38.0 ± 15.5	31.6 ± 1.1	24.5 ± 1.2
Zyśk et al. (2023)	Poland	Pilot	mixed	65	31	30.0–50.0	30.0–50.0	30.0–39.9	18.5–24.9

* Median (Q1-Q2); all other are presented as Mean ± SD

**USA**: Unites States of America; **UAE**: United Arab Emirates

### Analytical and Sampling Methods Used in Included Studies

As shown in Table 4, Enzyme-Linked Immunosorbent Assay (ELISA) was the most frequently used technique, appearing in the majority of studies either alone or in combination with other methods. Other techniques used in the different studies included Sodium Dodecyl Sulfate-Polyacrylamide Gel Electrophoresis (SDS-PAGE) [33], Matrix-Assisted Laser Desorption/Ionization Time-of-Flight (MALDI-TOF) [33], spectrometry [30], and Real-Time Polymerase Chain Reaction (PCR) [28], often combined with ELISA. Less commonly used methods included Inductively Coupled Plasma Mass Spectrometry (ICP-MS) [32], Enzyme Immunoassay (EIA), and Radioimmunoassay (RIA), with three studies applying multiple techniques [28, 30, 33]. Furthermore, several methods were utilized for saliva collection. These varied between unstimulated saliva, which is naturally secreted without external influence and was further categorized into methods such as spitting [31, 36, 40], passive drool [27–29, 33, 35, 37–39, 41, 42], and the use of a Salivette device [30, 32]; and stimulated saliva, where saliva secretion was stimulated by chewing on tasteless paraffin pellets [34].

**Table 4:** Summary of methods and analyzed salivary biomarkers in included published studies

Author (year)	Analyzed biomarkers	Analytical and sampling methods	Significant different biomarker levels (obese vs. control)*	Significant correlations (biomarkers to BMI)*	
Al-Ameri et al. (2024)	Thy-1	ELISA, unstimulated saliva	no significant difference was observed	-	
Al-Rawi et al. (2017)	Bacteria (*Fusobacterium spp.*,* P. gingivalis*,* T. forsythia*,* T. denticola*,* A. actinomycetemcomitans*), Resistin	ELISA, Real-Time PCR, unstimulated saliva (Passive drool)	*Fusobacterium spp.*,* P. gingivalis*,* T. forsythia* and Resistin higher in obese individuals	-	
Benedix et al. (2011)	Ghrelin	RIA, unstimulated saliva	no significant difference was observed	-	
Cheprasova et al. (2023)	Glucose, Cholesterol, Triglycerides, Total protein, chaperone activity, 8-OHdG, diene	Spectrometry, ELISA, unstimulated saliva (Salivette)	all measured biomarkers are higher in obese individuals	-	
Fejfer et al. (2017)	Total protein, 4-HNE, 8-isoP, AOPP, PC, 8-OHdG	ELISA, Assay, Mixed saliva (Spitting)	4-HNE, 8-isoP, AOPP, PC, 8-OHdG are higher in obese individuals and total protein is lower	-	
Junqueira et al. (2023)	Mn^2+^, Se^2+^, Sr^2+^, Zn^2+^, Cr^2+^, Cu^2+^, Fe^2+^, Ca^2+^, Mg^2+^	ICP-MS, unstimulated saliva (Salivette)	Ca^2+^ level higher in obese individuals	-	
Lamy et al. (2015)	sAA, ZAG, TMPRSS13, CST4	SDS-PAGE, Assay, MALDI-TOF, unstimulated saliva (Draining)	sAA, ZAG level higher in obese individuals	-	
Lehmann et al. (2018)	TNFα, TNFR1, MCP-1, PTX-3, total antioxidant capacity, catalase, superoxide dismutase, uric acid, VEGF, sICAM-1, PAI-1, E-selectin, leptin, adiponectin, serpin A12, resistin	Assays, ELISA, stimulated saliva (Paraffin pellet)	TNFα, TNFR1, MCP-1, PTX-3, sICAM-1, PAI-1, resistin, serpin A12 higher in obese individuals	-	
Lehmann et al. (2020)	TNFαR1, TNFαR2, PTX3, IL-15, MCP-1, sICAM-1, sCD40L	ELISA, unstimulated saliva	MCP-1, sICAM-1, TNFαR1, TNFαR2, PTX3, IL-15 higher in obese individuals and sCD40L is lower	-	
Matias et al. (2012)	Endocannabinoids (AEA, 2-AG) and N-acylethanolamines (PEA, OEA)	LC-MS/MS, PCR, unstimulated saliva (Spitting)	AEA, 2-AG, PEA, OEA higher in obese individuals	positive correlation between BMI and AEA, OEA	
Nigro et al. (2015)	Adiponectin	ELISA, unstimulated saliva	no significant difference was observed	-	
Öngöz Dede et al. (2016)	8-OHdG	EIA, unstimulated saliva	8-OHdG higher in obese individuals	-	
Ostrowska et al. (2020)	TNF-α, IL-8, sICAM1, calprotectin, MMP-9, MMP-2, TLR2	ELISA, unstimulated saliva	IL-8, calprotectin, MMP-2, TLR2 higher in obese individuals	positive correlation between BMI and TNF-α, IL-8, MMP-2	
Safabakhsh et al. (2022)	CRP, IL-6, IL-8, TAC	ELISA, unstimulated saliva (Spitting)	IL-6, IL-8 higher in obese individuals	positive correlation between BMI and IL-6, IL-8	
Soukup et al. (2012)	uric acid	Assay, unstimulated saliva (passive drool)	uric acid level higher in obese individuals	-	
Zyśk et al. (2023)	MMP-9, MMP-2, Resistin, IL-6, IL-1b	ELISA, unstimulated saliva	MMP-9, Resistin, IL-1beta higher in obese individuals	-	

* *p* < 0.05

### Salivary Biomarker Profiling in Individuals with Obesity from Included Studies

Tables 4 and 5 present a comprehensive overview of biomarker profiling in the included studies, highlighting significant differences between individuals with obesity and individuals in the control group. Table 4 outlines the methods and biomarkers analyzed, while Table 5 provides a summary of the different classes of salivary biomarkers, the specific biomarkers studied, the frequency with which these biomarkers showed significant differences between groups with obesity and control groups, and the clinical roles of these biomarkers.

**Table 5:** Summary of significant salivary biomarkers in individuals with obesity and corresponding study frequency

Biomarker category	Biomarker	Conducted studies	Clinical Role in Obesity-Related Disorders ^1,2,3^
DNA oxidation marker	8-Hydroxy-2’-deoxyguanosine (8-OHdG)	[29, 30, 37]	Predictor [43]
Ions	Calcium (Ca^2+^)	[31]	Predictor [44]
Enzymes	Salivary Alpha-Amylase (sAA)	[32]	Research stage
	Matrix Metalloproteinase-2 (MMP-2)	[38]	Research stage
	Matrix Metalloproteinase-9 (MMP-9)	[41]	Research stage
Inflammation markers	Interleukin-6 (IL-6)	[39]	Predictor [45]
	Interleukin-8 (IL-8)	[38, 39]	Predictor [46]
	Interleukin-15 (IL-15)	[34]	Research stage
	Resistin	[27, 33, 41]	Predictor [47]
	Interleukin-1 beta (IL-1beta)	[41]	Research stage
	Tumor Necrosis Factor-alpha (TNF-alfa)	[33]	Predictor [48]
	Tumor Necrosis Factor Receptor 1 (TNFR1)	[33, 34]	Research stage
	Tumor Necrosis Factor Receptor 2 (TNFR2)	[34]	Research stage
	Monocyte Chemoattractant Protein-1 (MCP-1)	[33]	Research stage
	Pentraxin-3 (PTX-3)	[33, 34]	Research stage
	Soluble Intercellular Adhesion Molecule-1 (sICAM-1)	[33, 34]	Predictor [49]
	Toll-Like Receptor 2 (TLR2)	[38]	Research stage
	Soluble CD40 Ligand (sCD40L)	[34]*	Research stage
Lipids	Cholesterol	[29]	Defining marker [50]
	Triglycerides	[29]	Defining marker [50]
*Lipid Peroxidation Products*	Diene	[29]	Research stage
	4-Hydroxynonenal (4-HNE)	[30]	Research stage
	8-isoprostanes (8-isoP)	[30]	Research stage
*Endocannabinoids*	Anandamide (AEA)	[35]	Predictor [51]
	2-Arachidonoylglycerol (2-AG)	[35]	Research stage
*N-acylethanolamines*	Palmitoylethanolamide (PEA)	[35]	Research stage
	Oleoylethanolamide (OEA)	[35]	Research stage
Metabolites	Uric acid	[40]	Research stage
Oral bacteria	*Fusobacterium* spp.	[27]	Research stage
	*P. gingivalis*	[27]	Research stage
	*T. forsythia*	[27]	Research stage
Proteins	Zinc-Alpha-2-Glycoprotein (ZAG)	[32]	Research stage
	Total protein	[29, 30] *	Research stage
	Plasminogen Activator Inhibitor-1 (PAI-1)	[33]	Research stage
	Calprotectin	[38]	Research stage
*Protein function*	Chaperone Activity	[29]	Research stage
*Protein oxidation markers*	Advanced Oxidation Protein Products (AOPP)	[30]	Research stage
	Protein Carbonyls (PC)	[30]	Research stage
Sugars	Glucose	[29]	Defining marker [52]

* Significantly lower in individuals with obesity individuals (*p* < 0.05); All others are significantly higher in individuals with obesity (*p* < 0.05)

**1**: Predictor: previously associated with an increased risk of developing obesity-related disorders (such as insulin resistance, metabolic syndrome, or cardiovascular disease)

**2**: Defining marker: used to diagnose or classify an obesity-related disorder based on established criteria

**3**: Research Stage: a biomarker under investigation; its clinical relevance is not yet established

In terms of oxidative stress biomarkers, 8-Hydroxy-2’-deoxyguanosine (8-OHdG) was reported as significantly elevated in individuals with obesity in multiple studies [30, 31, 38].

Among the ions measured, calcium (Ca^2+^) was found to be significantly higher in individuals with obesity [32]. Regarding enzymes, several studies observed significantly higher levels of salivary alpha-amylase (sAA), matrix metalloproteinase-9 (MMP-9), and matrix metalloproteinase-2 (MMP-2) in individuals with obesity, those in the control group [33, 39, 42].

A number of studies focused on inflammation markers, revealing that several were significantly elevated in individuals with obesity. These included interleukin-6 (IL-6) [40], interleukin-8 (IL-8) [39, 40], interleukin-15 (IL-15) [35], resistin [28, 34, 42], interleukin-1 beta (IL-1β) [33], tumor necrosis factor-alpha (TNF-α) [34], tumor necrosis factor receptor 1 (TNFR1) [34, 35], tumor necrosis factor receptor 2 (TNFR2) [35], monocyte chemoattractant protein-1 (MCP-1) [34], pentraxin-3 (PTX-3) [34, 35], soluble intercellular adhesion molecule-1 (sICAM-1) [34, 35] and toll-like receptor 2 (TLR2) [39]. Soluble CD40 ligand (sCD40L) was found to be lower in individuals with obesity [35].

Some studies investigated lipid biomarkers, including cholesterol, triglycerides, diene, 4-hydroxynonenal (4-HNE), and 8-isoprostanes (8-isoP), all of which were reported to be significantly elevated in individuals with obesity [30, 31]. One study measured endocannabinoids, including anandamide (AEA), 2-arachidonoylglycerol (2-AG), palmitoylethanolamide (PEA), and oleoylethanolamide (OEA) and found significantly higher levels in individuals with obesity [36]. Another study focused on the metabolite uric acid, reporting elevated salivary levels in individuals with obesity [41]. Concerning oral bacteria, one study identified significantly higher levels of *Fusobacterium spp*., *Porphyromonas gingivalis*, and *Tanerella forsythia* in individuals with obesity [28].

Various studies examined proteins, observing higher levels of several proteins in individuals with obesity, including zinc-alpha-2-glycoprotein (ZAG) [33], plasminogen activator inhibitor-1 (PAI-1) [34], calprotectin [39], chaperone activity [30], advanced oxidation protein products (AOPP) [31], and protein carbonyls (PC) [31]. However, there were conflicting findings for total protein, with one study reporting higher levels in individuals with obesity [30] and another reporting lower levels [31]. Finally, a single study measured glucose levels, which were found to be significantly elevated in individuals with obesity [30].

Of the biomarkers mentioned in Table 5, glucose, cholesterol, and triglycerides have been classified as defining markers, as they are used to diagnose or classify obesity-related disorders based on established criteria. Several others, 8-OHdG, Ca²⁺, IL-6, IL-8, resistin, TNF-α, and AEA, have been identified as predictors, as they have previously been associated with an increased risk of developing obesity-related conditions such as insulin resistance, metabolic syndrome, or cardiovascular disease. The remaining biomarkers were categorized as being in the research stage, indicating that they are still under investigation and their clinical relevance has not yet been established. This group includes multiple inflammatory markers, enzymes, lipid peroxidation products, and oral bacteria.

## Discussion

The current review presents an overview of the existing body of research on salivary biomarkers found in adults with MUO, highlighting key biomarkers that could potentially offer insights into alterations in salivary composition between individuals with MUO and those without MUO. Among the most frequently studied markers were those related to inflammation and oxidative stress, such as 8-OHdG [29–30, 37], IL-8 [38, 35], resistin [28, 39, 42], TNFR1 [34, 35], PTX-3 [34, 35], and sICAM-1 [34, 35], which were consistently elevated in individuals with MUO. Notably, IL-6 [40], IL-8 [39, 40], AEA, OEA [36], MMP-2, and TNF-α [34] were elevated in individuals with MUO and also correlated positively with BMI, further reinforcing the link between chronic inflammation and obesity.

These blood-derived biomarkers enter salivary tissues through transcellular pathways, such as passive and active transport, or via paracellular routes, including extracellular ultrafiltration [53] and selective transport alongside extracellular vesicles (EVs) which encapsulate biomarkers within lipid-bound vesicles (small, membrane-bound particles) and carry them from blood cells into saliva [54]. The elevated levels of these biomarkers were strongly linked to adipose tissue dysfunction, particularly in visceral fat, which acts as a metabolically active endocrine organ. Visceral fat releases pro-inflammatory cytokines, adipokines, and reactive oxygen species (ROS), generating a systemic inflammatory and oxidative environment that affects the entire body [55]. As visceral fat expands, it recruits and activates immune cells such as macrophages, leading to further production of ROS and inflammatory mediators. This excess ROS contributes to additional oxidative stress, cellular damage, and the elevation of biomarkers like 8-OHdG, which reflects oxidative DNA damage [56–57]. The cumulative effect of ROS not only damages cellular structures but also drives lipid peroxidation and protein dysfunction, which in turn drives the metabolic disturbances associated with obesity [58–59].

The chronic inflammation within visceral fat is accelerated by the secretion of ROS, cytokines and adipokines, such as IL-6, IL-8 and resistin, which maintain a low-grade inflammatory state that affects multiple systems within the body [60]. For example, IL-8 plays a key role in recruiting immune cells to sites of inflammation, contributing to systemic inflammation and insulin resistance. This relationship clarifies the positive correlation observed between IL-8 and BMI in some of the included studies in this review [39, 40]. Resistin, on the other hand, not only contributes to inflammation but also promotes insulin resistance [47], directly linking inflammation to metabolic dysregulation in individuals with obesity [61].

Moreover, the elevated levels of TNFR1 in obesity are a response to the increased production of TNF-α in hypertrophied visceral fat. This receptor mediates inflammatory pathways triggered by TNF-α, disrupting insulin signaling and promoting insulin resistance [62]. This dynamic creates a self-maintained cycle of inflammation and metabolic dysfunction, which likely accounts for the positive correlations between TNF-α and BMI found in one of the included studies [38]. Additionally, PTX-3, an acute-phase protein, rises in response to inflammatory stimuli from visceral fat and is closely linked to immune and inflammatory processes that contribute to insulin resistance [63].

Visceral fat-driven inflammation also impacts the vascular system, as evidenced by elevated levels of sICAM-1 and IL-8, both of which are markers of endothelial activation. This elevation reflects vascular dysfunction caused by chronic inflammation, which in turn promotes the adhesion and migration of immune cells to the endothelium. These processes contribute to the cardiovascular complications frequently observed in individuals with obesity [64].

Furthermore, endocannabinoids such as AEA and OEA have opposing effects on appetite and energy balance, possibly impacting obesity. AEA stimulates appetite and energy storage, with elevated levels being associated with increased visceral fat and metabolic dysregulation, which may play a role in the development of obesity. Conversely, OEA promotes satiety and fat oxidation, potentially countering obesity [65]. However, the study conducted by Matias et al. [36] showed a positive correlation between OEA and BMI. This could suggest that elevated OEA levels in individuals with obesity may be a compensatory response to suppress appetite dysregulation. This regulatory effect may weaken due to altered PPAR-α signaling, which is typically activated by OEA [66].

Interestingly, these review findings highlight that obesity is not merely a condition of excess fat accumulation but also a systemic disorder characterized by widespread metabolic and inflammatory disturbances. This systemic impact is reflected in metabolic biomarkers that could serve as indicators of obesity-related metabolic dysregulation. Among the defining markers, glucose, cholesterol, and triglycerides hold established clinical roles in the diagnosis of metabolic syndrome and obesity-related dyslipidemia. Elevated fasting glucose levels are a defining criterion for diabetes and metabolic syndrome, as recognized by the American Diabetes Association (ADA) and International Diabetes Federation (IDF). Similarly, altered lipid profiles, particularly elevated LDL cholesterol and triglycerides, along with decreased HDL cholesterol, are widely used as diagnostic criteria for cardiovascular risk assessment. The presence of these biomarkers in saliva suggests a potential for non-invasive metabolic screening [50, 52, 67, 68]. Although biomarkers such as 8-OHdG, IL-8, resistin, TNFR1, PTX-3, and sICAM-1 are closely associated with MUO and metabolic disorders, they have not yet been established as primary diagnostic biomarkers for obesity-tests in clinical practice. Rather than being used for direct diagnosis, these biomarkers offer valuable insights into the underlying metabolic disorders and inflammatory processes affecting individuals with MUO. They may serve as part of a biomarker panel aimed at tracking disease progression or evaluating responses to treatment. Therefore, further research is needed to determine their diagnostic potential, particularly their sensitivity and specificity in differentiating between obesity and other metabolic or inflammatory conditions. These markers could complement those mentioned earlier, helping to create a more comprehensive tool for early detection, diagnosis and monitoring obesity-related metabolic dysregulations. This suggests that relying on a single biomarker to capture the complexity of obesity-related metabolic dysregulation, including inflammation, and oxidative stress, is insufficient. Instead, a multi-biomarker approach is likely to provide a more accurate assessment of obesity progression in adults. By incorporating multiple biomarkers, this approach can enhance our understanding of the multifaceted nature of obesity and its complications.

### Limitations of Current Saliva Research

Despite the insights gained from this review, several gaps remain in the current body of research. Many studies lacked proper sample size justification or power analysis, which limits the reliability of their conclusions. Additionally, research on salivary biomarkers in individuals with obesity has been largely focused on inflammation and oxidative stress, while other biologically relevant categories such as oral microbiome, hormones, lipids, carbohydrates, vitamins, and ions seem less explored. Expanding the scope of research to include these biomarker categories could provide a more comprehensive picture of the salivary biomarker landscape in individuals with MUO.

Additionally, the lack of replication across studies investigating the same biomarkers makes it challenging to validate findings and establish consistency. A possible reason could be that current saliva biomarker research in the context of obesity is often focused on discovering novel biomarkers rather than validating existing knowledge. This highlights the need for reproducibility studies to confirm the diagnostic and prognostic value of previously reported biomarkers. Furthermore, although this review focused on salivary biomarkers related to obesity-induced systemic inflammation, obesity has also been associated with oral health alterations, including periodontal disease, microbial dysbiosis, and changes in salivary composition [69]. The potential bidirectional relationship between obesity and oral health remains an area for future investigation, particularly in understanding whether oral health status influences metabolic dysfunction in the context of obesity.

A critical limitation in current research is the lack of comprehensive validation studies comparing salivary biomarker levels with their corresponding blood levels, which remain the reference standard in clinical diagnostics. While saliva presents a promising non-invasive alternative for metabolic and inflammatory profiling, its diagnostic reliability depends on proper validation against blood-based biomarkers. Without this validation, the clinical applicability of salivary biomarkers remains uncertain, highlighting the need for large-scale studies to assess their sensitivity, specificity, and predictive value in obesity-related disorders. Establishing these correlations could clarify their potential role as complementary tools in obesity prevention and management.

Moreover, the absence of longitudinal studies further limits our understanding of how salivary biomarkers change over time in relation to obesity progression. Another methodological challenge is the lack of standardized saliva sampling methods, which introduces variability across studies and complicates the ability to draw consistent conclusions.

## Conclusion

This systematic review provided an overview of salivary biomarkers associated with MUO, including 8-OHdG, IL-6, IL-8, AEA, OEA, TNF-α, resistin, TNFR1, PTX-3, and sICAM-1, which reflect inflammation and oxidative stress. However, inconsistencies in saliva collection methods, a lack of validation against blood as a reference standard, and limited longitudinal studies present challenges to their diagnostic application. Future research should aim to address these gaps to further explore the potential of salivary biomarkers as early indicators of metabolic dysregulation associated with obesity and assess their clinical utility.

## Key References

- Ostrowska, L., Gornowicz, A., Pietraszewska, B., Bielawski, K., & Bielawska, A. (2020). Which salivary components can differentiate metabolic obesity?. PloS one, 15(6), e0235358. 10.1371/journal.pone.0235358.
- *Identifies key salivary biomarkers*,* including TNF-α and IL-8*,* as differentiators of metabolic obesity*,* highlighting their role in inflammation and BMI alternation.*
- Lehmann, A. P., Nijakowski, K., Swora-Cwynar, E., Łuczak, J., Czepulis, N., & Surdacka, A. (2020). Characteristics of salivary inflammation in obesity. Polish archives of internal medicine, 130(4), 297–303. 10.20452/pamw.15186.
- *Identifies key salivary inflammatory biomarkers associated with obesity*,* including TNF-R1*,* PTX3 and sICAM-1*,* highlighting their role in chronic systemic inflammation and their potential for non-invasive saliva testing.*
- Al-Ameri, H. W., Shetty, S., Rahman, B., Gopalakrishnan, A. R. K., Ismail, (A) A., & Acharya, A. (B) (2024). Evaluation of salivary Thy-1 in health, periodontitis, and obesity. Oral diseases, 30(4), 2670–2677. 10.1111/odi.14652.
- *Identifies Thy-1 as a novel salivary biomarker for obesity-related inflammation*,* highlighting its potential for non-invasive diagnostics and its association to both systemic and oral inflammation.*
- Safabakhsh, D., Jazaeri, M., Abdolsamadi, H., Abassi, E., & Farhadian, M. (2022). Comparison of salivary interleukin-6, interleukin-8, C - reactive protein levels and total antioxidants capacity of obese individuals with normal-weight ones. Romanian journal of internal medicine = Revue roumaine de medecine interne, 60(4), 215–221. 10.2478/rjim-2022-0013.
- *Investigates IL-6*,* IL-8*,* and C-reactive protein (CRP) in saliva*,* directly linking these pro-inflammatory markers to obesity*,* while also assessing total antioxidant capacity as a countermeasure to oxidative stress.*
- Junqueira, G. P., Junqueira-Franco, M. V. M., San Martin, R., Brandão, (C) F. C., Barbosa Júnior, F., Rocha, E. M., Chueire, F. B., & Marchini, J. S. (2023). Assessment of minerals in biological fluids in people with obesity: A pilot study. Journal of Trace Elements and Minerals, 3, Article 100,052. 10.1016/j.jtemin.2023.100052.
- *Analyzes trace mineral levels in saliva*,* identifying calcium as a key trace element correlated with obesity*,* expanding the scope of non-invasive biomarkers beyond inflammation markers.*
- Cheprasova, A. A., Popov, S. S., Pashkov, A. N., Verevkin, A. N., Mittova, V. O., & Shulgin, K. K. (2023). The intensity of free radical processes and chaperone activity in the saliva of patients with type 2 diabetes. BioMedicine, 13(2), 56–61. 10.37796/2211-8039.1407.
- *Examines oxidative stress markers in saliva in individuals with obesity and type 2 diabetes*,* highlighting the interaction between metabolic disorders and oxidative damage while introducing chaperone proteins as additional inflammatory markers.*

## Electronic Supplementary Material

Below is the link to the electronic supplementary material.

Supplementary Material 1

## Data Availability

No datasets were generated or analysed during the current study.
