# Supplementary figures and images for: Investigating the Salivary Biomarker Profile in Obesity: A Systematic Review
Source: Curr Obes Rep. 2025 Mar 28;14(1):25. doi: 10.1007/s13679-025-00618-y (PMC11953185; doi:10.1007/s13679-025-00618-y)

# Investigating the Salivary Biomarker Profile in Obesity: A Systematic Review

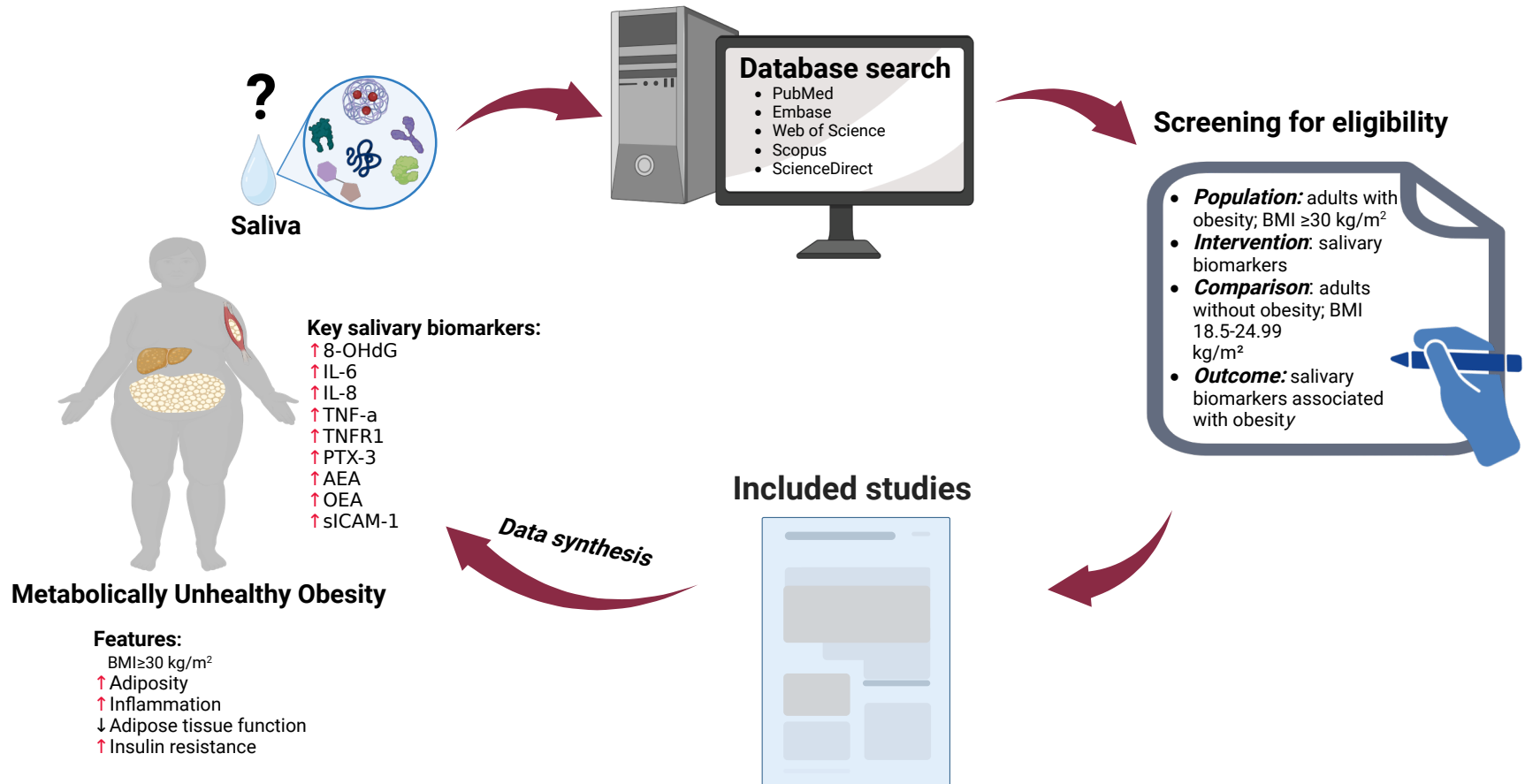

Supplement: Supplementary file 1 — Supplementary Material 1 [file 13679_2025_618_MOESM1_ESM.pdf]
